# Furunculosis

**Published:** 1911-06

**Authors:** L. Shingleton Smith


### FURUNCULOSIS.

BY

L. SHINGLETON SMITH, M.B. Cantab.

---

A FEW remarks from one who has been a victim to such a common yet troublesome disease as furunculosis may not be entirely without interest.

So much has been said and written, and Bier's cup has been so widely advertised for the treatment of boils, that I venture to ask whether those who believe so highly in its merits have only taken it from hearsay or have actually proved its efficiency either on their patients or themselves.

The beginning of my attack of boils coincided closely with my purchase of a mare—to be strictly accurate, the boil followed the mare by about a fortnight—and accordingly I put down the saddle as the exciting cause, though there is no doubt that I had been very run down for a month previously. The boils began to arrive with a quiet persistence, generally one at a time, and occasionally two together, but as soon as one had run its course the second would be well on its way ; at times they were so bad that I could neither ride the mare nor the bicycle, nor even drive, and had to give up work for a few days. Being thoroughly convinced by what I had read and heard of the utility of Bier's cupping treatment, and being desirous of ridding myself of such painful accessories, I vigorously applied the cup for an hour off and on both morning and evening, and sometimes midday when I could find the time, but to my surprise and discomfort without the looked-for success.

I applied the cup to the boils when first recognised, that is when they were not bigger than a large pin's head, but the application seemed not to have the slightest effect. The boil continued to grow, and in about three days the inguinal glands would be affected, and the boil having reached the size of a small saucer, would gradually subside and the slough separate, sometimes with a little pus.

If I applied the cup in its later stages the induration would spread under the edge of the cup, and the application become most painful.

The only way in which the cup seemed to be of any benefit was after the free incisions (of which I had well over a dozen), when the matter and slough came away perhaps a little more readily than would otherwise have been the case. Various other treatments were adopted ; but in spite of all—Bier's cupping, hot Jeyes' baths, sulphur and mercurial inunctions, and tonics—the boils kept the even tenor of their way, in nowise abating.

It was then, after much prompting, that I determined to try what vaccine therapy could do, and accordingly from some pus sent them the Clinical Research Association isolated and grew the staphylococcus pyogenes aureus, and in a short time sent me a dozen phials, containing different doses, 20, 25, 50, 100, 150, 250 and 500 million cocci. A few hours after the first dose of 20 millions had been injected an extraordinary reaction, both locally and constitutionally, appeared. The boil, at that time getting well, became exceedingly tender and more painful than it had been at all, and after a few hours got better again. On the next day I felt a bit feverish, and the temperature rose to 103. I felt and looked so ill that Dr. A. J. Wright, who was very kindly doing my work and helping me with the injections, thought I was in for a bad illness. However, at 6 p.m. I arose and had a good meal, feeling perfectly well. After this increasing doses were given regularly about once a week, and at first slight reactions, both locally and constitutionally, appeared, but later no reaction at all. The boils gradually became fewer and less severe, and finally disappeared altogether for the space of six months.

All the boils during the latter period were painted with liq. iod. fort., and when detected and painted whilst quite small simply withered up and gave no further trouble, and if allowed to develop to say the size of a shilling, they spread no farther, came to a head, and the slough separated in a comparatively short time.

After the boils had ceased to come there continued to appear pimples, which shortly became pustular, and with painting withered up, which clearly shows that the resistance had been increased, inasmuch that those same pimples two months earlier would have shown no sign of pus, but spread as an indurated phlegmonous mass affecting the inguinal glands. Six months later another boil came in the same region, and presuming that the same organism was at work, an injection of 250 million was given; and following this there came at least a dozen commencing boils, all of which were painted with liq. iod. fort., and all of them aborted.

Whilst the injections were continued, at first at regular intervals, the boils steadily diminished in numbers, and there was no sudden outbreak of them as appeared six months later after the injection of a single dose of 250 million cocci. From this it appears that the immunity acquired had to a great extent disappeared, inasmuch as a boil appeared, and after the large injection a negative phase was manifested by the sudden abundant crop of small boils.

Three months later a small boil began, and another three months after that, both of which were aborted by the application of liq. iod. fort.

Although Bier's cupping has been highly lauded in the treatment of furunculosis, in my experience (a most painful one) the application of liq. iod. fort. is far more efficacious, and this I put down to the following reasons.

Firstly, the iodine application produces hyperæmia in the skin and not in the fascia and subcutaneous tissue, as I think the cup is more apt to do, especially in the case of boils which are situated for the most part in the soft parts, such as the neck, back, and gluteal region, when the skin is readily lifted up and drawn into the cup without very much stretching.

Secondly, the iodine application is constant and always acting, whether the patient be busy or otherwise.

Thirdly, the iodine destroys the germs in the surrounding skin.

In all acutely hyperæmic states of the skin, whether they be due to sunburn, chilblains, toxic rashes, infectious fevers, or slight scalds, peeling more or less invariably follows, but I have never seen the application of Bier's cup followed by any tendency to desquamation.

This may in part be due to the inefficient method of carrying out the application, for to keep the part congested one must be constantly busy applying the cup, a method which forbids one to do anything else; and if in a region where one cannot apply it with the help of a looking-glass, then it must prevent someone else from doing any work. It is quite impossible for a doctor on his round to apply the cup with any likelihood of doing good. In the case of iodine one can always apply it oneself; two and occasionally three coats may be necessary, but invariably within a short time the boil begins to mend.

Therefore we may presume that vaccine therapy is the most valuable constitutional remedy we have, but the immunity acquired is only temporary, whilst liq. iod. fort. is the most valuable and convenient local remedy.

